# Identification of Spliceogenic Variants beyond Canonical GT-AG Splice Sites in Hereditary Cancer Genes

**DOI:** 10.3390/ijms23137446

**Published:** 2022-07-04

**Authors:** Vita Šetrajčič Dragoš, Ksenija Strojnik, Gašper Klančar, Petra Škerl, Vida Stegel, Ana Blatnik, Marta Banjac, Mateja Krajc, Srdjan Novaković

**Affiliations:** 1 Department of Molecular Diagnostics, Institute of Oncology Ljubljana, SI-1000 Ljubljana, Slovenia; vsetrajcic@onko-i.si (V.Š.D.); gklancar@onko-i.si (G.K.); pskerl@onko-i.si (P.Š.); vstegel@onko-i.si (V.S.); 2 Biotechnical Faculty, University of Ljubljana, SI-1000 Ljubljana, Slovenia; ablatnik@onko-i.si; 3 Cancer Genetics Clinic, Institute of Oncology Ljubljana, SI-1000 Ljubljana, Slovenia; kstrojnik@onko-i.si (K.S.); mbanjac@onko-i.si (M.B.); mkrajc@onko-i.si (M.K.); 4 Faculty of Medicine, University of Ljubljana, SI-1000 Ljubljana, Slovenia

**Keywords:** aberrant splicing, deep intronic variants, in silico prediction tools, hereditary cancer syndrome, pseudoexon, reclassification, RNAseq, spliceogenic, spliceogenic variant, splice variant, VUS, VUS reclassification

## Abstract

Pathogenic/likely pathogenic variants in susceptibility genes that interrupt RNA splicing are a well-documented mechanism of hereditary cancer syndromes development. However, if RNA studies are not performed, most of the variants beyond the canonical GT-AG splice site are characterized as variants of uncertain significance (VUS). To decrease the VUS burden, we have bioinformatically evaluated all novel VUS detected in 732 consecutive patients tested in the routine genetic counseling process. Twelve VUS that were predicted to cause splicing defects were selected for mRNA analysis. Here, we report a functional characterization of 12 variants located beyond the first two intronic nucleotides using RNAseq in *APC*, *ATM*, *FH*, *LZTR1*, *MSH6*, *PALB2*, *RAD51C*, and *TP53* genes. Based on the analysis of mRNA, we have successfully reclassified 50% of investigated variants. 25% of variants were downgraded to likely benign, whereas 25% were upgraded to likely pathogenic leading to improved clinical management of the patient and the family members.

## 1. Introduction

RNA splicing is a complex process catalyzed by the spliceosome in which introns are removed from pre-mRNA and protein-coding exons are ligated together, creating mature mRNA. The spliceosome must accurately recognize the intron/exon boundaries to generate a functional gene transcript. Multiple *cis*-acting splicing motifs are crucial during the intron/exon recognition process, including consensus donor’ (5′) and acceptor (3′) splice site (ss), branch site, polypyrimidine tract, splicing enhancers, and silencers [1]. Variants situated within the *cis*-acting splicing motifs can cause erroneous recognition of splicing motifs by the spliceosome, which can lead to disruption of the open reading frame causing a nonfunctional protein or activation of nonsense-mediated decay pathway [2,3]. According to the American College of Medical Genetics and Genomics and Association for Molecular Pathology (ACMG/AMP) variant classification guidelines, disruption of highly conserved GT-AG splicing motif (±1 or 2 splice sites) is considered very strong evidence for variant’s pathogenicity [4]. Therefore, it is fairly simple to classify such variants as likely pathogenic just based on their predicted effect on splicing. However, splicing impairment can be triggered by variants situated beyond the first two intronic nucleotides. Variants of all kinds, both exonic and intronic, can lead to an out-of-frame transcript by multiple mechanisms. Variants can abolish natural ss, create de novo ss, activate cryptic ss, destroy branch sites, or disrupt splicing enhancers or silencers. To predict variants’ impact on splicing, multiple in silico prediction tools have been developed. Bioinformatics tools are frequently most accurate around the consensus ss [1,5]. Although in silico splicing tools are highly valuable in predicting variants’ spliceogenity, alone, they are not sufficient to reclassify a variant (e.g., from variants of uncertain significance (VUS) to likely pathogenic) as computational predictions are considered supportive evidence for pathogenicity according to ACMG/AMP guidelines. The computational prediction should be confirmed with RNA-based assays. Without the experimental data, most of the non-truncating variants in hereditary cancer genes are classified as uncertain significance [6,7]. In this study, we have bioinformatically evaluated VUSs obtained from 732 patients who have undergone genetic testing for hereditary cancer syndromes at our clinic. Patients harboring VUS with predicted impact on splicing were eligible for additional RNA analysis. Using our approach previously published [8] using long-range PCR and deep sequencing, we have functionally characterized 12 variants positioned outside of GT-AG splicing motif that were predicted to cause splicing abnormalities in *APC*, *ATM*, *FH*, *LZTR1*, *MSH6*, *PALB2*, *RAD51C*, and *TP53* genes. Furthermore, the study aimed to explore the impact of RNA analysis on variant classification.

## 2. Results

**Figure 1:**
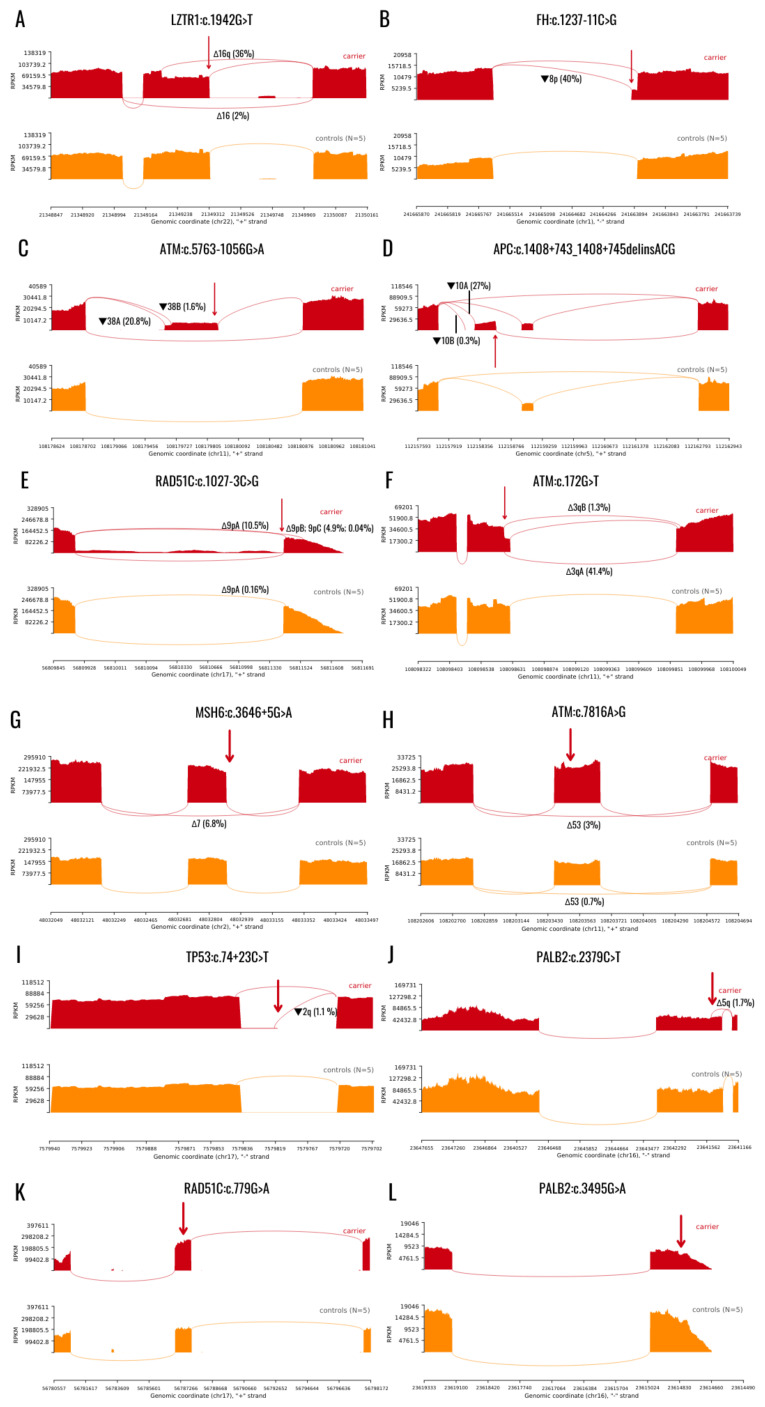
Sashimi plots representing variant’s impact on splicing. Variant (**A**) **u***LZTR1*:c.1942G>T caused complete splicing impairment (Class 3S), variants (**B**) *FH*:c.1237-11C>G; (**C**) *ATM*:c.5763-1056G>A; (**D**) *APC*:c.1408+743_1408+745delinsACG; (**E**) *RAD51C*:c.1027-3C>G; (**F**) *ATM*:c.172G>T; (**G**) *MSH6*:c.3646+5G>A; (**H**) *ATM*:c.7816A>G; (**I**) *TP53*:c.74+23C>T; (**J**) *PALB2*:c.2379C>T caused incomplete or uncertain splicing impairment (Class 2S) and variants (**K**) *RAD51C*:c.779G>A; (**L**) *PALB2*:c.3495G>A have not impaired splicing. Red arrows represent variant’s location. Red graphs depict carriers, orange graphs depict control samples. RPKM—reads per kilobase of exon model, represents the number of mapped reads in the sample.

In this study, we have determined the spliceogenicity of 12 variants located outside of the 5′ and 3′ GT-AG dinucleotides using RNAseq. To our knowledge, except for one variant that has been previously evaluated with mRNA analysis (*PALB2*:c.2379C>T), the remaining 11 have not been previously assessed for their impact on splicing [7]. Seven variants are novel as they have not been described in the literature or any variant databases. All variants have been classified as VUS based on their pathogenic in silico prediction and/or low frequency in the population database GnomAD or because they have not yet been functionally assessed. Three variants that were examined were deep intronic, five were deep exonic, and four variants were located in the consensus donor or acceptor ss. One variant showed complete splicing alteration, nine variants caused partial or uncertain splicing aberration, and two variants had no impact on mRNA splicing. Complete splicing aberration (Class 3S) was considered when the variant did not produce any full-length transcript. Incomplete or uncertain splicing aberration was considered when the variant produces aberrant and full-length transcript or we have been unable to determine whether the variant produces any full-length transcript or not (Class 2S). Lastly, for variants in which no splicing aberration was detected, we have grouped them into variants without impact on splicing (Class 1S). For a complete HGVS description of the variant’s impact on splicing, see Table 1. Sashimi plots created from alignment files are depicted in Figure 1.

### 2.1. Variants Causing Complete Splicing Impairment (Class 3S)

***LZTR1*:c.1942G>T** variant is located in the ultimate nucleotide of exon 16 and abolishes the natural donor ss of intron 16. Variant produces two out-of-frame aberrant transcripts: Δ16q a cryptic 5′ss activation leading to a primarily expressed 112 nt deletion of exon 16 (36% of reads) and a slightly expressed event Δ16 a complete skipping of exon 16 (2% of reads). Both abnormal transcripts were not present in control samples (Figure 1A). At position c.1942, only wild type allele was observed.

### 2.2. Variants Causing Incomplete or Uncertain Splicing Impairment (Class 2S)

***FH*:c.1237-11C>G** variant introduces a de novo 3′ss in between nucleotides c.1237-10 and c.1237-11. De novo ss outcompetes the native acceptor ss and triggers the retention of 10 intronic nucleotides of intron 8 (▼8p). The aberrant transcript was present in 40% of reads and was absent in controls (Figure 1B).

***ATM*:c.5763-1056G>A** variant enhances the 5′cryptic ss between cDNA positions c.5763-1055 and c.5763-1054 in intron 38. Two cryptic 3′ss are activated to create 137 and 120 nucleotides long pseudoexons ▼38A and ▼38B, respectively. Primarily expressed transcript ▼38A was expressed in 20.8%, whereas ▼38B was expressed in 1.6% reads. Both pseudoexons are predicted to create frameshift and premature stop codons. None of the aberrant transcripts were present in controls (Figure 1C).

***APC*:c.1408+743_1408+745delinsACG** variant creates a novel 5′ss between the positions c.1408+744 and c.1408+745 in intron 10. Two different cryptic 3′ss have been activated and, therefore, the variant produced two aberrant mature mRNA transcripts with 98 (▼10A) and 143 (▼10B) nucleotides (nts) pseudoexon inclusions. ▼10A was predominantly expressed (27% of reads) compared to ▼10B (0.3% of reads). Both pseudoexons disrupt the open reading frame and introduce premature stop codons. None of the aberrant transcripts were present in control samples (Figure 1D).

***RAD51C*:c.1027-3C>G** variant is located in the ultimate intron of the *RAD51C* gene. It alters the acceptor ss of intron 8 and triggers the creation of multiple aberrant mRNA transcripts: most prevalent was the Δ9pA inframe deletion of the first 6 nt of exon 9 (10.5% of reads), followed by Δ9pB, a frameshift deletion of first 52 nt of exon 9 (4.9% of reads). Additionally, the variant caused intron retention; however, it was not possible to determine from NGS data whether full intron or partial intron has been retained in the mature mRNA. Two control samples also harbored a transcript Δ9pA, but in an extremely low fraction (0.16% reads), suggesting it is a normally present alternative splicing event with low expression (Figure 1E). Indeed, the Δ9pA event has been previously reported as a naturally-occurring lowly expressed event [11].

***ATM*:c.172G>T** was demonstrated to inactivate native donor ss and generate a de novo 5′ss motif located 15 nt from native ss, causing a frameshift deletion of the last 16 nucleotides of exon 3 (Δ3qA) and a deletion of 16 last nucleotides of exon 3 and first 10 nucleotides of exon 4 (Δ3qB). However, when visualizing alignment files, we have observed that the mutated allele T appeared in normally spliced reads, suggesting that a portion of the full-length transcript is generated even with the mutated allele. Δ3q transcript was present in 41% of transcripts, full-length transcript with T allele at c.172 positions was estimated to produce 8% of transcripts, and the remaining was the full-length transcript generated by wild-type G allele. Control samples did not harbor the aberrant transcript (Figure 1F).

***MSH6:c.3646+5G>A*** variant alters the native donor ss of intron 7, causing in-frame exon 7 skipping (Δ7) in 6.8% of reads. Controls did not harbor Δ7 event (Figure 1G).

***ATM*:c.7816A>G** variant is predicted to create a de novo donor ss 28 bp upstream of natural ss. We have not detected the anticipated 5′ss take over; however, we have observed a whole exon 53 skipping slightly more expressed than in five control samples (3% vs. 0.7%-ranging from 0.3 to 1.3%). Additionally, when bam files were analyzed, wild type A allele was present in 57% of reads and the mutated G allele in 43%, indicating a slight reduction of the mutated allele (Figure 1H).

***TP53*:c.74+23C>T** variant is predicted to create a novel 5′ss in intron 2. A minimally expressed 21bp intron retention event (▼2q) was detected in 1.1% and 1.7% of reads in two unrelated patients but not in controls (Figure 1I).

***PALB2*:c.2379C>T** variant is a deep exonic variant located in exon 5, located 135 bp 5′ of native donor ss. According to bioinformatics analysis, the variant creates a strong de novo donor ss. We have observed that the novel donor ss motif can outcompete the natural ss; however, it creates abnormal transcript in a very low percentage (1.7% and 0.3% in two unrelated individuals). The majority of transcripts harboring the c.2379C>T variant were not aberrantly spliced and have produced a full-length transcript. The aberrant transcript was not present in controls (Figure 1J).

### 2.3. Variants Not Interrupting Splicing (Class 1S)

Introduction of de novo acceptor ss was bioinformatically predicted for deep exonic variant ***RAD51C*:c.779G>A**, whereas ***PALB2*:c.3495G>A** was predicted to introduce a de novo donor ss. Furthermore, the two variants did not disrupt splicing and have only produced normal full-length transcripts (Figure 1K,L).

## 3. Discussion

ACMG/AMP guidelines are becoming a gold standard for interpreting germline variants in clinical laboratories worldwide. Variants are classified into five pathogenicity tiers based on 28 pieces of evidence of different weights (e.g., functional data, phenotype and family history data, computational prediction, variant frequency in control populations, etc.) [4]. However, ACMG/AMP guidelines do not take into consideration fewer penetrant phenotypes such as hereditary cancer syndromes. Multiple difficulties are faced when interpreting variants causing hereditary cancer syndromes: segregation studies are hindered due to the presence of phenocopies and also due to the fact that cancer risk varies between males and females in certain syndromes [12,13]. As clinical and family history data is difficult to associate with variant’s pathogenicity, multiple ACMG/AMP pieces of evidence cannot be applied to genes involved in oncogenesis. Therefore, clinical molecular diagnostics laboratories deal with a great number of rare variants that cannot be conclusively interpreted as clinically significant or not and are therefore placed in a category of variants of uncertain significance (VUS). To clarify the pathogenicity of VUS, functional studies that investigate variants’ effect on gene products are of extreme importance. A well-documented mechanism of hereditary cancer syndrome development are variants in susceptibility genes that interrupt RNA splicing. It is estimated that 15–25% of DNA variants in hereditary cancer genes cause mis-splicing [5]. Therefore, to decrease the VUS burden, we have bioinformatically evaluated all novel VUS in our cohort and selected those that were predicted to cause splicing defects. Patients harboring such VUS were invited to provide a blood sample for RNA analysis and functional characterization of 12 variants located beyond the first two intronic nucleotides using RNAseq was performed. Based on the analysis of mRNA, we could detect splicing impairment for 10 out of 12 variants. To categorize variants, which showed an impact on splicing, we have modified the ACMG/AMP usage of PS3 criteria based on a workflow suggested by Nix et al. [14], Feliubadaló et al. [15], and Garret et al., 2021 [16]. PS3-strong evidence was applied if the alternative allele did not produce any normal transcript. As intronic variants are not a part of an mRNA molecule, it is not possible to determine whether the variant produces any normal transcript or not, without an exonic tag SNP. Therefore, for intronic variants for which we observed splicing impairment but were without an exonic tag SNP, PS3 was downgraded to moderate evidence of pathogenicity (PS3-m).

We were able to apply PS3 as strong evidence of pathogenicity for one variant only. ***LZTR1*:c.1942G>T** primarily caused partial exon 16 deletion (36%) and secondary induced less expressed whole exon skipping (2%). Importantly, at position c.1942, only the wild-type allele was observed, indicating that the variant causes complete splicing impairment, and no full-length transcript is formed from the T allele. Even though the patient is a heterozygous carrier of the variant, the aberrant transcripts were detected in less than 50%, indicating that nonsense-mediated decay (NMD) has been activated due to the creation of a premature stop codon. Additionally, the variant is rare and has been observed in a patient with schwannomatosis, which is associated with *LZTR1* deficiency. Splice-altering variants have been previously described as causative in patients with schwannomatosis [17]. Hence, ACMG/AMP criteria PS3, PM2, PP3, and PP4 were applied, classifying the variant as likely pathogenic.

We were able to apply PS3-moderate for five variants, which were shown to disrupt splicing, but lacked exonic tag SNP. Two variants ***APC*:c.1408+743_1408+745delinsACG** and ***ATM*:c.5763-1056G>A** were determined to activate pseudoexon inclusion of intron 13 (27% aberrant reads) and intron 38 (22.4% aberrant reads) respectively (Table 1). None of the variants harbor an exonic SNP to determine whether any full-length transcript is formed from a mutated allele; therefore, PS3-moderate was applied. *ATM*:c.5763-1056G>A caused pseudoexon inclusion event ▼38A (137 bp) of intron 38 that has been described before, but it was caused by a different, well-studied pathogenic variant *ATM*:c.5763-1050A>G, located 6 nucleotides upstream of our variant. The *ATM*:c.5763-1050A>G was proven to create incomplete splicing damage, which results in mild ataxia-telangiectasia phenotype in homozygous carriers [18]. Unfortunately, the variant *ATM*:c.5763-1050A>G was not present in our in-house patient database to perform mRNA analysis and compare the level of expression of intron inclusion events. Our variant *ATM*:c.5763-1056G>A was detected in a heterozygous state in a breast cancer patient. Heterozygous pathogenic variants in the *ATM* gene are associated with an increased risk of estrogen receptor positive breast cancer [19,20]. Due to reduced penetrance of heterozygous *ATM* variants, PP4 was not applied, leaving the *ATM*:c.5763-1056G>A variant classified as VUS (PS3-m, PM2-supp, and PP3). Indeed, we hypothesize that our variant *ATM*:c.5763-1056G>A could be pathogenic, causing a similar effect as *ATM*:c.5763-1050A>G; however, additional studies and clinical data from homozygous or compound heterozygous carriers are needed to confirm our speculation.

***APC*:c.1408+743_1408+745delinsACG** located in intron 13 was detected in a patient with colon polyposis (38 tubular adenomas), which are associated with *APC* pathogenic variants. Additionally, pseudoexon inclusion events in intron 13 of the *APC* gene have been previously described as pathogenic [21]. The variant was reclassified to likely pathogenic with PS3-m, PM2, PP3, and PP4 criteria. The variant ***FH*:c.1237-11C>G** created a novel acceptor ss, causing intron retention predicted to create an out-of-frame transcript. The aberrant transcript was detected in a high percentage (40%) in a patient with leiomyomatosis. Loss-of-function variants, including splicing variants in the *FH* gene, have been associated with hereditary leiomyomatosis and renal cell cancer (HLRCC) syndrome [22]. Considering these facts, our variant was reclassified as likely pathogenic by applying PS3-m, PM2, PP3, and PP4 classification criteria. Clinical features of the patients and their family history are described in Table 2.

***PALB2*:c.2379C>T, *PALB2*:c.3495G>A**, and ***TP53*:c.74+23C>T** variants were all predicted to create a de novo ss, stronger than consensus ss. Functionally, they generated an extremely low splicing impairment (all below 2% of reads). The newly generated splice sites could not entirely outcompete the natural ss, possibly due to the absence of *cis*-acting motifs needed for exon identification. As splicing impairment was insignificant for all three variants and they are predicted synonymous or intronic, we were able to downgrade them to likely benign. The effect on splicing was previously evaluated for the *PALB2*:c.2379C>T variant in the study published by Karam et al. [7]. Similar to our results, they have not detected impaired splicing and have also reclassified the variant from VUS to likely benign using ACMG/AMP criteria: BS3 (well-established in vitro functional study shows no effect on splicing) and PP3 (computational tools predict splicing impairment). Importantly, according to ACMG/AMP guidelines, a combination of evidences BS3 (in vitro functional studies show no impact on splicing) and PP3 (in silico tools support a deleterious effect on the gene product) (1 strong benign plus 1 supporting pathogenic evidence) is not sufficient to reach likely benign classification. Oddly, it is possible to classify a new rare silent variant as likely benign solely based on benign computational prediction, applying two supporting pieces of evidence, BP4 and BP7, without performing any functional studies. Therefore, a silent variant for which in silico tools predict splicing impairment, but the functional mRNA study disproves the prediction cannot be placed in the class 2 category without additional benign evidence. Whereas a silent variant for which only in silico tools predict no effect on splicing can be categorized as likely benign. To our knowledge, this classification dispute has not been raised in the literature before. For that reason, similarly to Karam et al. [7], we have utilized BS3 as stand-alone evidence for silent variants, where no significant impact on splicing was observed, to be able to classify them as likely benign. Additionally, to apply BS3 as stand-alone evidence, the nucleotide should not be evolutionary conserved.

However, five variants remained classified as VUS as there were not enough data for more precise interpretation and classification. First, variant ***RAD51C*:c.779G>A** p.(Arg260Gln) did not exhibit a splicing anomaly (Figure 1K); however, the variant also results in an amino acid change from arginine to glutamine. Arginine at position 260 is conserved up to a zebrafish [23]. Therefore, ACMG/AMP evidences PM2 and PP3 were applied, without BS3, as there is no functional data available for missense’s impact on protein function. Second, ***ATM*:c.7816A>G** p.(Ile2606Val) was shown to have only a minimal effect on mRNA splicing (Figure 1H), making the missense change the most important effect of the variant. Moreover, protein in silico tools predict its benign role, but no protein or phenotypic studies are available to confirm it; therefore, the variant remains classified as VUS (ACMG/AMP evidence applied: PP3).

Third, ***MSH6*:c.3646+5G>A** variant caused in-frame deletion of exon 7 (Δ7). Inframe deletions are not a subject of NMD and are expected to create approximately 50% of the aberrant transcript. The percentage of Δ7 transcript was only 6.8%, which is below the expectations, implying this is an incomplete ss variant. It has not yet been established what proportion of incorrectly spliced transcripts will confer pathogenicity for Lynch syndrome genes. For example, previous studies have observed incomplete splicing aberration for variant *MSH2*:c.1275A>G (35% aberrant versus 15% full-length transcript) and have classified it as VUS [24,25,26]. But, with further clinical evidence, some laboratories have eventually classified it as likely benign [24,27]. Therefore, we classify this variant as VUS (ACMG/AMP evidence applied: PM2, PM4, PP3) and additional protein studies should be performed to conclusively classify *MSH6*:c.3646+5G>A variant.

Fourth, the ***ATM*:c.172G>T** variant produced a high amount of abnormal transcripts Δ3qA and Δ3qB (43%), but we could detect mutated allele T in 8% of full-length transcripts, signifying incomplete splicing impairment. Incomplete splicing variants causing reduced penetrance in hereditary cancer syndromes have been reported in the literature [28,29]. Incomplete ss variants are predicted to cause the reduction of the wild-type protein and might manifest as later disease onset or milder phenotype. However, the estimation of how much the variant reduces the penetrance and what kind of surveillance should be offered to the patient is not yet fully recognized. Therefore, as there is no agreed threshold when leaky ss variant is indeed pathogenic, we have conservatively classified *ATM*:c.172G>T as VUS applying PM2-supp, PP3, and PS3-m classification criteria.

Fifth, ***RAD51C*:c.1027-3C>G** variant is located in the last intron of *RAD51C* and it reduces the strength of natural acceptor ss, causing multiple aberrant transcripts. The most expressed was the in-frame deletion of 2 amino acids, followed by the deletion of 52 bp and the least expressed was the deletion of 55 bp, both leading to a frameshift. Additionally, we have detected an intron inclusion event, which we could not precisely determine the length of and its level of expression. Therefore, additional studies such as long-read sequencing should be performed to precisely determine the consequence of *RAD51C*:c.1027-3C>G variant, leaving the variant classified as VUS, using ACMG/AMP evidences PM2, PP3, PS3-m. Long-read sequencing could improve the classification of the variant as it enables full-length transcript analysis and quantification, which is not achievable with the short-read sequencing approach used in this study. Of note, a different variant, located 1 bp 3′ of our variant (c.1027-2A>G), has been previously classified as pathogenic on the assumption it alters splicing, but no RNA studies have been performed to our knowledge [30].

Although RNAseq can often resolve a variant’s clinical classification, protein functional studies coupled with clinical data are still fundamental and, in some cases, crucial for a comprehensive understanding of the variant’s effect. Especially, variants causing incomplete splicing defects can preserve enough functional transcript to maintain normal protein function. Therefore, caution when interpreting RNA results is needed. For instance, previously published variant *BRCA2*:c.426-12_426-8del, which was initially classified as likely pathogenic due to detection of exon 5 skipping, was later reclassified as benign as the splicing aberration was actually incomplete. The benign classification was additionally supported by clinical data [7,14]. Indeed, additional evidence, such as comprehensive functional studies that measure the effect of variants on multiple layers of gene function, represents an enormous advantage in variant interpretation in the clinical setting [31,32].

However, based on the analysis of mRNA, we were able to reclassify 6 out of 12 variants (50%). Three (25%) were reclassified as likely benign and three (25%) as likely pathogenic. A similar but more comprehensive study conducted by Karam et al. [7], in which 56 VUS in hereditary cancer predisposition genes were evaluated using RNAseq, have reached an 88% VUS reclassification rate. Of all VUS, 47% were reclassified as pathogenic and 41% as benign. In an additional RNA study, 33% of analyzed VUS exhibited abnormal splicing, which implies their pathogenic role [33]. In comparison, the diagnostics yield of our study was similar to the study conducted by Wai et al. [33], whereas it was significantly lower than in the study performed by Karam et al. [7]. The lower reclassification yield might be due to our stringent rule for applying PS3 as strong evidence, which results in a lower number of reclassifications to likely pathogenic. In case we applied PS3 as strong evidence for all variants that interrupted splicing, even variants with incomplete splicing impairment, one could misclassify variants as likely pathogenic.

Additionally, we were very cautious in reclassification of missense VUS by not applying the BS3 rule (in vitro functional study shows no effect on gene product), which caused a lower rate of likely benign reclassifications.

In summary, we have confirmed that variants beyond the canonical GT-AG ss can cause aberrant splicing in hereditary cancer syndromes. Based on the functional characterization of VUS, using RNA sequencing, we have successfully reclassified 50% of investigated variants. 25% of our variants were upgraded to likely pathogenic leading to improved clinical management of the patient and the family members.

## 4. Materials and Methods

### 4.1. Ethical Approval

The present study was approved by the Institutional Review Board of the Institute of Oncology Ljubljana (permission no. OIRIEK00937) and by the National Medical Ethics Committee of the Republic of Slovenia (permission no. 0120-339/2019/5). All procedures followed in the present study were, therefore, in accordance with the ethical standards of the responsible committees on human experimentation (institutional and national) and the Helsinki Declaration of 1975, as revised in 2013. Individual written informed consent from the participant for the publication of this report was obtained as well as the institutional informed consent form for treatment, which included consent to use the patient’s data, materials, and/or test results for research purposes. All patients underwent genetic counseling before and after the genetic test.

### 4.2. Patients and Germline Genetic Screening

Patients referred to the germline genetic testing for hereditary cancer syndromes at the Cancer Genetics Clinic, Institute of Oncology Ljubljana, had undergone genetic counseling. In total, 732 patients underwent NGS panel testing using hybrid capture library preparation with TruSight Cancer or TruSight Hereditary Cancer Panel (Illumina, San Diego, CA, USA) enrichment oligos performed as described before [34,35,36]. VUS, classified according to ACMG/AMP criteria, identified in genes associated with the patient’s phenotype and family history were evaluated for their impact on splicing using in silico prediction tools. A total of 12 VUS in *APC*, *ATM*, *FH*, *LZTR1*, *MSH6*, *PALB2*, *RAD51C*, and *TP53* (detected in 16 unrelated patients whose personal and family history is described in Table 2) met the criteria for RNA evaluation (the criteria description is written below in Section 4.3). The blood samples for RNA analysis were obtained from 14 patients (patients 4 and 11 were not available for additional blood draw). Reclassification reports for all patients recruited in this study have been sent out to the referring clinical geneticist. Cascade testing of the relatives was advised, where the variant was upgraded to likely pathogenic.

All diagnoses stated in Table 2 were verified in the Cancer Registry of the Republic of Slovenia, a national registry of mandatory reported cancer cases. Family history of cancer in Table 2 is reported for first- and/or second-degree relatives.

### 4.3. Criteria for the Variant to Be Included in Functional Characterization by RNAseq

Bioinformatic tools for splicing prediction NNSplice, MaxEntScan, GeneSplicer, SpliceSiteFinder-like (SSF) (all included in Alamut Visual software), SpliceAI [37] and SPICE were used [38]. SpliceAI Lookup web interface was used and the Δ score was calculated with a distance of ±50 bp from the variant. For the variant to be predicted causative, the SpliceAI Δ score had to be higher than 0.2. Variants with at least two in silico tools predicting a creation of novel 5′ or 3′ss regardless of predicted ss strength were selected for mRNA analysis. Additionally, variants causing a reduction of natural ss strength, either one in silico tool predicted complete loss of ss or MaxEntScan showed a reduction of 15% and SSF reduction of 5%, were chosen for mRNA analysis. The criteria were adapted by Houdayer et al. [5], Thomassen et al. [39], and Santos et al. [40]. Splicing prediction results for all variants are described in Supplementary Table S1.

Based on the position of the variant relative to the ss, the variants were divided into three groups:

(1) variants located in the consensus 3′ or 5′ss (consensus donor ss (5′ss) is defined as DNA motif spanning from the last three exonic to the first eight intronic nucleotides and acceptor ss (3′ss) spanning from the last twelve intronic till the first two exonic nucleotides) [1]
(2) deep intronic variants,
(3) deep exonic variants,

Variants that were positioned outside of the defined consensus donor or acceptor ss were considered deep intronic or deep exonic variants.

### 4.4. RNAseq

The blood samples of 16 patients were collected into Tempus Blood RNA Tube (ThermoFisher, Waltham, MA, USA). Total RNA was isolated from whole blood using Tempus™ Spin RNA Isolation Kit (ThermoFisher). RNA quantity was measured using Qubit™ RNA HS Assay Kit (ThermoFisher). cDNA synthesis was performed with SuperScript™ IV VILO™ Master Mix (ThermoFisher) using 100 ng of total RNA.

RNAseq was performed using an in-house developed approach as previously described [8]. In short, primer pairs aligning to the 5′ and 3′ UTR regions of gene of interest were designed. cDNA was amplified using long-range PCR using LongAmp^®^ Taq 2X Master Mix (New England Biolabs, Ipswich, MA, USA). NGS library was prepared using a transposon that cleaved PCR products using Nextera XT according to the manufacturer’s instructions (Illumina). The library was paired-end sequenced (2 × 121 cycles) on NextSeq 550 (Illumina). FASTQ files were generated using the bcl2fastq2 tool (Illumina). FASTQ files were aligned to the hg19 genome build using the STAR aligner [41]. Split reads and splicing events were obtained from the OutSJ.tab file, produced by the STAR aligner. Sashimi plots were generated using the rmats2sashimiplot tool.

Transcript quantification was calculated as previously described by Davy et al. [42]. Briefly, for each junction, the ratio of mutated/normal junction counts was calculated and presented as a percentage.

Aberrant transcripts are described using the following symbols: Δ—partial or whole exon skipping, ▼—intron insertion, p—acceptor shift, q—donor shift. If multiple aberrant transcripts occurred within the same exon/intron, subsequent letters were added to the event. For example, two acceptor shifts causing partial exon 3 deletions were described as Δ3pA and Δ3pB.

As control samples (N = 5), we used patients’ samples in which only clearly benign variants were detected for studied genes. Primer pair sequences can be obtained upon request.

RefSeq sequences were used: NM_000038.4 (*APC*)–exon numbering 2-18 was used (LRG-specific), NM_000051.3 (*ATM*), NM_000143.3 (*FH*), NM_006767.3 (*LZTR1*), NM_000179.2 (*MSH6*), NM_024675.3 (*PALB2*), NM_058216.1 (*RAD51C*), NM_000546.5 (*TP53*).

## Figures and Tables

**Table 1:** Variants’ effect on mRNA splicing (detected with RNAseq) and final variant classification.

cDNA Variant	Variant Type	Splicing Event Description	Effect on mRNA Splicing	Predicted Protein Change	% Aberrant Transcript Carrier	mean % Aberrant Transcript Controls (N = 5)	Splicing Classification [5]	ACMG/AMP Evidences	ACMG/AMP Classification after RNAseq
***APC*:c.1408+743_1408+745delinsACG** intronic	DI	▼10A ▼10B	r.1408_1409ins1408+647_1408+744 r.1408_1409ins1408+602_1408+744	p.(Gly471Serfs*60) p.(Gly470Alafs*13)	27% 0.3%	ND ND	2S	PM2, PP3, PS3-m, PP4	4-LP
***ATM*:c.172G>T** missense	DE	Δ3qA Δ3qB	r.171_185del r.171_195del r.172g>u	p.(Trp57*) p.(Trp57Cysfs*14) p.(Asp58Tyr)	41.4% 1.3% 8%	ND ND ND	2S	PM2-supp, PP3, PS3-m	3-VUS
***ATM*:c.5763-1056G>A** intronic	DI	▼38A ▼38B	r.5762_5763ins5762+985_5763-1055 r.5762_5763ins5762+1002_5763-1055	p.(Arg1921Serfs*6) p.(Arg1921Serfs*12)	20.8% 1.6%	ND ND	2S	PM2-supp, PP3, PS3-m	3-VUS
***ATM*:c.7816A>G** missense	DE	Δ53	r.7789_7927del r.7816a>g	p.(Asp2597Lysfs*3) p.(Ile2606Val)	3% 43%	0.7% ND	2S	PP3	3-VUS
***FH*:c.1237-11C>G** intronic	3′ss	▼8p	r.1236_1237ins1237-1_1237-10	p.(Ile413Serfs*5)	40%	ND	2S	PM2, PP3, PS3-m, PP4	4-LP
***LZTR1*:c.1942G>T** missense	5′ss	Δ16q Δ16	r.1831_1942del r.1786_1942del r.1942g>u	p.(Val611Alafs*4) p.(Glu596Alafs*4) p.(Gly648Cys)	36% 2% ND	ND ND ND	3S	PM2, PP3, PS3, PP4	4-LP
***MSH6*:c.3646+5G>A** intronic	5′ss	Δ7	r.3557_3646del	p.(Glu1187_Gly1216del)	6.8%	ND	2S	PM2, PP3, PM4	3-VUS
***PALB2*:c.2379C>T** synonymous	DE	Δ5q	r.2378_2514del r.2379c>u	p.(Gly793Aspfs*2) p.(Gly793=)	1.7%; 0.3% 44%; 50%	ND ND	2S	BS3-SA, BS1, PP3	2-LB
***PALB2*:c.3495G>A** synonymous	DE	/	no aberrant transcript detected r.3495g>a	/ p.(Ser1165=)	ND 48%	ND ND	1S	BS3-SA, BS1 PP3	2-LB
***RAD51C*:c.1027-3C>G** intronic	3′ss	Δ9pA Δ9pB Δ9pC	r.1027_1032del r.1027_1078del r.1027_1081del	p.(Pro343_Gln344del) p.(Pro343Lysfs*4) p.(Pro343Valfs*3)	10.5% 4.9% 0.04%	0.16% ND ND	2S	PM2, PP3, PS3-m	3-VUS
***RAD51C*:c.779G>A** missense	DE	/	no aberrant transcript detected r.779g>a	/ p.(Arg260Gln)	ND 45%	ND ND	1S	PM2, PP3	3-VUS
***TP53*:c.74+23C>T** intronic	DI	▼2q	r.74_75ins74+1_74+21	p.(Leu26*)	1.1% and 1.7%	ND	2S	BS3-SA	2-LB

Splicing classification is adapted by Houdayer et al. [5], Class 1S: no effect on splicing, Class 2S: partial effect on splicing, or it could not be determined whether it is partial splicing defect or complete defect due to the lack of exonic tag SNP, Class 3S: severe impact on splicing, the mutant allele does not produce a full-length transcript., DI: deep intronic, DE: deep exonic, 3′ss: consensus 3′ ss, 5′ ss: consensus 5′ ss ND: not detected, LB: likely benign, VUS: variant of uncertain significance, LP: likely pathogenic, PM2: the variant is rare in GnomAD database, PP3: in silico tools predict the deleterious effect on gene product, PS3: in vitro, the functional assay showed a deleterious effect on gene product, PS3-m: PS3 moderate strength, PP4: patient’s phenotype is associated with a specific disease, BS3-SA: stand-alone evidence for variants that do not impair splicing, BS1: variant allele frequency is greater than expected for a disorder, PM4: variant causes in-frame deletion,. *ATM* and *TP53* variants were classified according to [9,10], respectively.

**Table 2:** Personal and family history of cancer in patients that have undergone mRNA analysis.

Patient Number	cDNA Variant	Personal History of Tumors (Age at Diagnosis)	Family History of Tumors (Age at Diagnosis)	Another Pathogenic Variant Detected in a Patient.
**1**	*APC*:c.1408+743_1408+745delinsACG	CP (51)	RC (79)	-
**2**	*ATM*:c.5763-1056G>A	Bil BC (61)	GC (60); UC (52)	-
**3**	*ATM*:c.7816A>G	BC (41)	-	*BRCA2*:c.4139_4140dup p.(Lys1381Leufs*8)
**4**	*ATM*:c.7816A>G	Bil BC (49,52)	-	-
**5**	*ATM*:c.172G>T	MM (53), BC (68)	3 NHL (49,76,80); 2 MM (51,79), BC (84); GCT (16)	-
**6**	*FH*:c.1237-11C>G	L (37)	BC (53), MM (52)	-
**7**	*MSH6*:c.3646+5G>A	BC (37)	-	-
**8**	*PALB2*:c.2379C>T	BC (78)	BC (41); GC (82)	-
**9**	*PALB2*:c.2379C>T	BC (46)	BC (42)	*CHEK2*:c.444+1G>A p.?
**10**	*PALB2*:c.3495G>A	BC (59)	2 BC (37,40); LaC (58)	-
**11**	*RAD51C*:c.1027-3C>G	OC (49)	-	-
**12**	*RAD51C*:c.1027-3C>G	OC (74)	NHL (46), NSCLC (54)	-
**13**	*RAD51C*:c.779G>A	BC (43)	BC (41), OC (41)	*BRCA1*:c.3718C>T p.(Gln1240*)
**14**	*TP53*:c.74+23C>T	MBC (42), NSCLC (61)	SCLC (60), LiC (59); CRC (54), GC (75), PrC (72); CC (46), LaC (57)	-
**15**	*TP53*:c.74+23C>T	BC (49)	2 BC (64,84); PrC (70)	-
**16**	*LZTR1*:c.1942G>T	SCH (54)	NA	-

BC: breast cancer; Bil: bilateral; CC: cervical cancer; CP: colon polyposis; CRC: colorectal cancer; GC: gastric cancer; GCT: germ cell tumor; L: leiomyomatosis; LaC: laryngeal cancer; LiC: liver cancer; MBC: male breast cancer; MM: malignant melanoma; NA: not available; NHL: non-Hodgkin’s lymphoma NSCLC: non-small cell lung cancer; OC: ovarian cancer; PrC: prostate cancer; RC: renal cancer; SCH: schwannomatosis; SCLC: small cell lung cancer; UC: uterine cancer.

## Data Availability

The data presented in this study are available upon request from the corresponding author. The data are not publicly available due to patients’ privacy.

## Supplementary Materials

The following supporting information can be downloaded at: https://www.mdpi.com/article/10.3390/ijms23137446/s1.
